# Meta-analysis of the acute effects of anodal transcranial direct current stimulation on athletic performance

**DOI:** 10.3389/fphys.2025.1631905

**Published:** 2025-08-12

**Authors:** Mi Jiang, Yang Liu, Xu Gao

**Affiliations:** ^1^ School of Physical Education, Northeast Normal University, Changchun, China; ^2^ School of Physical Education, Nanchang Normal University, Nanchang, China

**Keywords:** transcranial direct current stimulation (tDCS), athletic performance, endurance, motor cortex, meta-analysis

## Abstract

**Objective:**

Systematically evaluate the acute effects of anodal transcranial direct current stimulation (a-tDCS) on athletes’ sport-specific performance and identify the optimal stimulation parameters and target brain regions for enhancing sport-specific performance.

**Methods:**

Search PubMed, Web of Science, CNKI, Wanfang, and other databases to include randomized controlled trials studying the effects of anodal tDCS on sports performance in healthy athletes. Use a random-effects model to calculate the standardized mean difference (SMD), assess heterogeneity, and evaluate influencing factors. Additionally, conduct three subgroup analyses: (1) based on stimulated brain areas (M1, PFC, TC, CB); (2) based on different sports performance domains (endurance, strength, precision skill tasks, competitive-collaborative skills) for cluster analysis; (3) tDCS protocol parameters (current intensity and stimulation duration).

**Results:**

This study included 31 articles, covering 473 athletes. The meta-analysis results showed that the acute effect of a-tDCS significantly improved athletes’ specific sports performance, with a moderate effect size (SMD = 0.39, 95% CI = 0.23–0.54, p < 0.001). Subgroup analysis revealed that M1 stimulation had the most consistent effect (SMD = 0.32, 95% CI = 0.15–0.48, p < 0.001), followed by PFC stimulation (SMD = 0.39, 95% CI = 0.03–0.76, p = 0.03). a-tDCS significantly enhanced athletes’ endurance performance (SMD = 0.46, 95% CI = 0.20–0.72, p < 0.001) and competitive-collaborative skill tasks (SMD = 0.45, 95% CI = 0.10–0.80, p = 0.01). Analysis of stimulation parameters indicated that a moderate current intensity of 1.6–2.0 mA (SMD = 0.38, p < 0.001) and a stimulation duration of 16–20 min (SMD = 0.45, p < 0.001) were the optimal protocols for enhancing sports performance.

**Conclusion:**

The acute effects of a-tDCS significantly enhance athletes’ endurance and competitive-collaborative skill performance, particularly when targeting the M1 and PFC regions. The optimal stimulation protocol involves a moderate current intensity (1.6–2.0 mA) and duration (16–20 min). Future research should further optimize stimulation parameters and explore long-term effects to enhance the application of a-tDCS in sports training.

**Systematic Review Registration:**

https://www.crd.york.ac.uk/prospero/display_record.php?RecordID=103158, identifier CRD42025103158.

## 1 Introduction

With the integration of neuroscience and sports science, transcranial direct current stimulation (tDCS), due to its simplicity, low cost, and high safety, has become one of the most widely studied methods among non-invasive brain stimulation (NIBS) techniques in high-level athletic training research (Bhattacharya et al., 2022; Chang et al., 2022; Hazime et al., 2017). tDCS involves the application of a low-intensity direct current (typically 1–2 mA) via scalp electrodes to modulate cortical excitability in targeted brain regions, thereby influencing sensorimotor pathways and behavioral outputs (Nitsche and Paulus, 2000). Although the precise neurophysiological mechanisms underlying the effects of tDCS remain incompletely understood, several prevailing hypotheses suggest that alterations in cortical excitability (Notturno et al., 2014), enhancement of synaptic plasticity (Stagg and Nitsche, 2011), modulation of regional cerebral blood flow (Jones et al., 2015; Paquette et al., 2011; Wachter et al., 2011), and reorganization of functional brain network connectivity (Meinzer et al., 2012) are involved.

Rather than relying on a single area, motor activity engages a broad network of brain regions known as the motor neural network. This study focuses on the effects of a-tDCS on four key brain areas: the primary motor cortex (M1), dorsolateral prefrontal cortex (PFC), temporal/temporoparietal cortex (TC), and cerebellum (CB). tDCS can be applied using either anodal (a-tDCS) or cathodal (c-tDCS) stimulation, with the former typically enhancing cortical excitability by depolarizing neurons, and the latter reducing excitability via hyperpolarization (Jamil et al., 2017). However, some studies have reported reversed effects, where a-tDCS decreases excitability and c-tDCS increases it (Batsikadze et al., 2013; Hassanzahraee et al., 2020a; 2020b), suggesting that outcomes depend on electrode placement, individual brain anatomy, and stimulation parameters. Generally, a-tDCS lowers the action potential threshold and increases motor-evoked potentials (MEPs), thus improving motor performance, while c-tDCS can inhibit overly active neural circuits and is sometimes used in treatments such as epilepsy (Sudbrack-Oliveira et al., 2021).

The selection of sport-specific tasks is critical for evaluating the effects of tDCS. In contrast to abstract tasks, such as the sequence reaction-time task (Ehsani et al., 2016) sport-specific tasks offer higher ecological validity, thereby providing a more accurate reflection of actual performance in competitive environments. Sport-specific tasks facilitate the activation of higher potential through the motor transfer effect (Abernethy et al., 2005), capitalizing on the neuromuscular adaptations acquired through an athlete’s long-term training. Based on the theoretical framework of sports science and the neurophysiological mechanisms of tDCS, the study categorized sports performance into four subgroups: endurance, strength, competitive-collaborative skills, and precision skills. Endurance and strength reflect physical demands, involving cardiorespiratory function and explosive power output, respectively, primarily regulated by M1 (motor control) and dlPFC (fatigue tolerance). Competitive-collaborative skills and precision skills involve cognitive and fine motor functions, relying on the autonomic and coordination functions of M1, dlPFC, TC, and CB (Bompa and Buzzichelli, 2019).

Over the past decade, a substantial number of observational studies have emerged globally. However, the findings have been inconsistent. Existing literature reports that tDCS can improve cognitive and motor functions in healthy populations (Angius et al., 2018a; Lu et al., 2021; Teymoori et al., 2023; Zhiqiang et al., 2023) and clinical patients (Bornheim et al., 2022; González-Zamorano et al., 2024; Lee et al., 2019; Wiegand et al., 2019). In athletes, tDCS has shown both positive and negative effects. Several studies have reported that anodal tDCS (a-tDCS) can enhance athletic performance. For instance, Okano et al. (2015) found that M1 stimulation improved peak power output in cyclists, and Pollastri et al. (2021) confirmed that dlPFC stimulation enhanced performance in a 15 km time trial Chen et al., (2021) and Zhang et al. (2024) reported that M1 stimulation improved basketball shooting accuracy and jump height, respectively. However, Lattari et al. (2020) found that M1 stimulation had no significant effect on strength performance, and Mesquita et al. (2020) reported that bilateral M1 stimulation even reduced kicking frequency in taekwondo athletes. These inconsistencies may arise from differences in inter-cortical competitive inhibition, stimulation target areas, stimulation parameters, and task types.

Previous meta-analyses on tDCS primarily focused on the general population (Machado et al., 2019a); Winker et al., 2024) or single outcome measures (Lattari et al., 2018; Shyamali Kaushalya et al., 2022). This study systematically analyzes the acute effects of a-tDCS on athletes’ sport-specific performance by integrating Chinese and English literature, conducting subgroup analyses of brain regions, performance domains, and stimulation parameters, aiming to identify optimal protocols and provide a scientific basis for optimizing athletic training.

## 2 Research methods

### 2.1 Registration

This study follows the PRISMA guidelines to ensure comprehensive and transparent reporting of the methods and results (Moher et al., 2009). The study protocol has been registered in the PROSPERO database (Registration number: CRD420251031580).

### 2.2 Literature search strategy

This study searched six Chinese and English databases, including PubMed, Web of Science, CNKI, and Wanfang. English search terms included: (“tDCS” OR “transcranial Direct Current Stimulation”) AND (“athlete” OR “player” OR “sport” OR “athletic performance” OR “physical performance”). In Chinese, the search terms were: (“Transcranial Direct Current Stimulation/经颅直流电刺激” OR “tDCS” AND (“Motor Skills/运动技能” OR “Motor Performance/运动表现” OR “Motor Learning/运动学习” OR “Strength and Conditioning/体能”) TS=(“Athletes/运动员” OR “Sports Training/体育训练” OR “Competitive Sports/竞技运动”)). Search terms in both languages were adjusted for linguistic and cultural differences in academic terminology while maintaining semantic equivalence to ensure comprehensive coverage of relevant studies. To reduce cultural or regional study selection bias, the search strategy included global (English) and regional (Chinese) databases, supplemented by manual searches of references from included studies to identify additional relevant articles. The search strategy was developed by Mi Jiang (MJ) and Yang Liu (YL) and reviewed and optimized by Xu Gao (XG) to ensure consistency and comprehensiveness across databases.

### 2.3 Inclusion and exclusion criteria

The literature inclusion criteria were established based on the PICOS principle. The criteria for study inclusion were defined as follows. Population: Athletes, defined as healthy individuals regularly engaged in competitive or professional sports training, with no restrictions on training level and free from injuries or diseases; no limitations on gender or ethnicity. Intervention: anodal transcranial direct current stimulation, high-definition transcranial direct current stimulation (HD-tDCS), or Halo Sport headset. Comparison: sham stimulation or no stimulation control group. Outcomes: Comparison: Sham stimulation or no-stimulation control group. Outcomes: Sport-specific task performance, such as time to exhaustion (s) for endurance tasks, jump height (cm) for strength tasks, shooting accuracy (%) for precision skills, or serve speed (m/s) for competitive-collaborative skills. Study Design: Randomized controlled trials (RCTs) with crossover or parallel designs.

We excluded studies involving participants with diseases, rehabilitation populations, or animal studies; studies not reporting outcomes related to motor skills or physical performance; qualitative descriptions, conference abstracts, or reviews lacking data support; studies with incomplete or unextractable raw data; and conference abstracts, book chapters, or short articles in languages other than English or Chinese.

### 2.4 Literature screening process

The retrieved citation information was imported into EndNote 21 software for de-duplication. Data management was carried out using Excel, which included extracting key bibliographic details (including title, first author, journal name, publication year, and participant characteristics). The eligibility of studies was assessed by two independent reviewers, Xu Gao (XG) and Yang Liu (YL), in a standardized manner from 18 April 2025, to 20 April 2025. Following the previously defined inclusion criteria, all articles identified using the search strategy were screened based on their titles and/or abstracts. If sufficient information was not available to assess eligibility, the full-text version was obtained. Full texts of all potentially relevant studies were reviewed by XG and YL to ensure compliance with the inclusion criteria. Discrepancies between XG and YL were resolved through discussion, with Mi Jiang (MJ) acting as the arbiter to reach a final consensus. If any of the included articles lacked sufficient information or data, the authors were contacted to obtain additional details.

### 2.5 Data extraction and risk of bias assessment

Two researchers independently conducted literature screening and data extraction. Initial screening was performed based on titles and abstracts, with disagreements resolved through discussion according to inclusion criteria until consensus was reached. Full-text reviews were then conducted, and references were traced for additional relevant studies. Researchers collected the following information: (1) basic study details (author, year, sample size); (2) tDCS parameters (stimulation site, current intensity, duration); (3) outcome measures and their measurement methods; (4) study design characteristics.

To clearly present the data, studies in the table were sorted by publication year (from earliest to latest) to reflect temporal trends in tDCS research; for highlighting effect differences, studies were secondarily grouped by effect size. Additionally, the GRADE (Grading of Recommendations Assessment, Development, and Evaluation) method was used to assess the certainty of evidence for primary outcomes, evaluating five dimensions: risk of bias, inconsistency, indirectness, imprecision, and publication bias. GRADE assessments were independently conducted by MJ and YL, with disagreements resolved through discussion and arbitration by XG to ensure reliability.

The Cochrane Risk of Bias Tool was used to assess the quality of the included studies, evaluating seven dimensions of study quality, including random sequence generation, allocation concealment, and blinding procedures. When blinding was clearly described as double-blind for both experimenters and participants, the study was rated as “low risk.” If only single-blind or unclear blinding was mentioned, the study was rated as “unclear.” MJ and YL independently rated the studies, with a Kappa consistency coefficient of 0.85, indicating high agreement. When disagreements occurred, discussions were held based on the evaluation criteria, with Xu Gao (XG) acting as the arbiter until consensus was reached.

### 2.6 Data analysis

Statistical analysis was conducted using RevMan 5.4. Cluster analysis was performed by grouping studies according to stimulation brain regions (M1, PFC, TC, CB), athletic performance domains (endurance, strength, precision skill tasks, and competitive-collaborative skill tasks), and tDCS parameters (current intensity, stimulation duration) to explore sources of effect heterogeneity. In cases where a study involved multiple motor tasks, the task most representative of the competitive performance of the respective athlete population was selected (see Table 1 for details). For example, backward movement jumps were measured by vertical height in centimeters, endurance performance by time to exhaustion in seconds, shooting accuracy by the percentage of successful hits, and competitive-collaborative skill tasks by specific performance metrics, such as serve speed in meters per second. For tasks assessing reaction time or timed races, the mean result values were multiplied by −1 to ensure all intervention effects were aligned in the same direction (i.e., shorter reaction times indicate better performance). Due to the varying units of outcome measures across the included studies, the standardized mean difference (SMD) was used as the effect size. Referring to the Cochrane Handbook for Systematic Reviews, heterogeneity was quantitatively assessed using the p-value and I^2^ statistic. I^2^ represents the level of heterogeneity among studies, ranging from 0% to 100%. When I^2^ ≤ 50%, a fixed-effect model was used for meta-analysis; otherwise, a random-effects model was applied, and subgroup analysis was conducted to identify and determine the mediating variables causing heterogeneity. A p-value <0.05 was considered statistically significant; otherwise, no statistical significance was inferred. Given the diversity in athletic performance, stimulation parameters, and participant characteristics, undetected heterogeneity might exist. Therefore, this study adopted a random-effects model to pool data (Barili et al., 2018) for a more robust estimation of the overall effect of a-tDCS on specific athletic performance. To verify the reliability of the results, sensitivity analysis was performed using a fixed-effect model (Rendina-Gobioff, 2006), sequentially removing individual studies to observe changes in the pooled effect size and assess whether any single study significantly influenced the overall results. The results showed that the effect sizes and confidence intervals of both models were highly consistent, demonstrating a certain degree of robustness.

**TABLE 1:** Basic characteristics of the included studies.

Study	Design	Sample (F-female,M-male)	Participants	Anode (A) Cathode (C)	Current intensity (mA)	Stimulation duration (min)	Sport performance	Indicator description	Outcome
Okano et al. (2015)	Single-blind, crossover	10 (all M)	cyclists	A—left TC(F3) C—right supraorbital area (Fp2)	2	20	Peak power output (PPO) on a bicycle power meter	Peak Power (w)	a-tDCS improved PPO compared to sham
Kamali et al. (2019b)	Double-blind, RCT	16(8F,8M)	pistol-shooters	A—CB2 C—dlPFC	2	20	Shooting score on 10 m shooting range	Target score (points)	a-tDCS improved Shooting score compared to sham
Kamali et al. (2019a)	Double-blind, crossover	12 (all M)	bodybuilders	A1— M1(Cz,C1,C2)A2—left TC (T3) C1—right shoulder C2—left shoulder	2	13	Short-term Endurance Index (SEI): AMRAP for knee extension and 30% 1RM	SEI is calculated by multiplying the weight (30% of 1RM) by the number of consecutive exercises. (%)	a-tDCS improved SEI compared to sham
Mesquita et al. (2019)	Single-blind, crossover	19(7F,12M)	taekwondo black belts	A1—right M1(C3)A2—left M1(C4) C1—right shoulder C2—left shoulder	1.5	15	Frequency speed of kick test (FSKT)	Number of kicks (times)	No significant difference in total number of kicks between a-tDCS and sham
Lattari et al. (2020)	Double-blind, crossover	10 (all M)	Athletes	A—Bilateral M1(Cz) C—right orbitofrontal cortex (Fp2)	2	20	Countermovement Jump (CMJ) test	CMJ height (cm)	a-tDCS improved CMJ height compared to sham
Mesquita et al. (2020)	Single-blind, crossover	12(4F,8M)	taekwondo black belts	A1—right M1(C3)A2—left M1(C4) C1—right shoulder C2—left shoulder	1.5	15	Progressive specific taekwondo test (PSTT)	Peak kick frequency (kicks/min)	No significant difference for peak kicking frequency between a-tDCS and sham
Chen et al. (2021)	Single-blind, crossover	13 (all M)	basketball players	A—M1(Cz) C—M1(C5,C6)Halo Sport	2	20	Countermovement Jump (CMJ) test	Jump height (m)	a-tDCS improved CMJ height compared to sham
Pollastri et al. (2021)	Double-blind, RCT	8 (all M)	cyclists	A1—left dlPFC(F3)A2—right dlPFC(F4) C1—Fp1/F7/C3 C2—Fp2/F8/C4 (HD-tDCS)	1.5	20	15-km TT bicycle dynamometer	15 km cycling time (seconds)	a-tDCS improved TT compared to sham
Penna et al. (2021)	Single-blind, crossover	10 (all M)	swimmers	A—left TC (T3) C— ipsilateral shoulder	2	30	800 m swimming TT	800mTT(s)	No significant difference in 800 m swimming TT between a-tDCS and sham
da Silva Machado et al. (2021)	Single-blind, crossover	12 (all M)	Professional endurance athletes (7 cyclists and 5 rowers)	A—M1(Cz) C—inion (HD-tDCS)	2	20	Time to exhaustion (TTE) test with 80% PPO on cycle ergometer	Exhaustion time (s)	No significant difference in TTE between a-tDCS and sham
Nikooharf Salehi et al. (2022)	Single-blind, crossover	15 (all M)	swimmers	A—left dlPFC (F3) C—right supraorbital area (Fp2)	2	20	50 m freestyle swimming in a state of fatigue	50 m swimming time (s)	a-tDCS improved freestyle swimming performance compared to sham
Liang et al. (2022)	Single-blind, crossover	8 (all F)	elite female rowing athlete	A—M1(Cz) C—M1(C5,C6) Halo Sport	2.2	20	5 km TT on rowing ergometer	5 km rowing machine test (s)	No significant difference in 5 km TT on rowing ergometer between a-tDCS and sham
Gallo et al. (2022)	Double-blind, crossover	11 (all M)	cyclists	A1—left dlPFC(F3)A2—right dlPFC(F4) C1—Fp1/F7/C3 C2—Fp2/F8/C4(HD-tDCS)	1.5	20	2 km TT on cycle ergometer	2 km cycling time (s)	a-tDCS improved 2 km TT compared to sham
Park et al. (2023)	Single-blind, crossover	13 (all F)	Professional female volleyball player	A—M1(Cz) C—M1(C5,C6) Halo Sport	2	20	Spiking consistency test	Spiking consistency (km/h)	a-tDCS improved Spiking consistency test compared to sham
Fortes et al. (2022)	Double-blind, crossover	19 (all F)	Amateur swimmer	A—left oPFC(Fp1) C—right (Fp2)	2	30	Minimum Strength Test in Tethered swimming	Minimum Strength Test in tethered swimming (N)	a-tDCS improved endurance performance compared to sham
Zhuangzhuang (2024)	Double-blind, crossover	10 (all F)	High-level golfer	A—M1 (C3) C—supramarginal region (Fp3)	2	20	The hitting effect of the No. 1 wooden club (landing distance)	Distance from landing point (yards)	a-tDCS improved the hitting effect of female high-level golferscompared to sham
Zhiqiang et al. (2023)	Double-blind, RCT	20 (all F)	Rhythmic gymnast	A—M1 (C3,C4) C—dlPFC(Fp1,Fp2)	2	20	Modified CTSIB score under the condition of soft ground with eyes closed	Modified CTSIB Balance Score (points/10 s)	a-tDCS improved the Modified CTSIB scorecompared to sham
Moreira et al. (2023)	Double-blind, crossover	8 (all F)	Professional female basketball player	A—left dlPFC (F3) C—right dlPFC (F4)	2	20	The task of shooting from behind the three-point line	Number of shots required to reach 10 three-point shots (times)	No significant difference in three-point line shooting task between a-tDCS and sham
Luo et al. (2023)	Double-blind, crossover	20(8F,12M)	Rock climber	A—M1(C3,C4) C—M1(C3,C4) Halo Sport	2	20	The explosive force of pulling down one arm of the right arm	Right arm single arm pull-down explosive force (W)	a-tDCS improved the explosive force of pulling down one arm of the right armcompared to sham
Anoushiravani et al. (2023)	Single-blind, crossover	17 (all M)	Gymnast	A—M1(C3,C4); CB C—M1(Fp1,Fp2)	2	20	Broad jump test (BJT)	Distance (cm)	a-tDCS improved the BJTperformance compared to sham
Etemadi et al. (2023)	Double-blind, RCT	14 (all M)	Endurance athlete	A1— M1(Cz)A2—left dlPFC(F3) C1—left shoulder C2—supraorbital region (AF8)	2	20	The exhaustion time TTE of a 30-min power bicycle	TTE (minutes)	a-tDCS improved endurance performance compared to sham
Kamali et al. (2023)	Double-blind, crossover	14 (all M)	cyclists	A—M1 C—lumbar spinal cord (T12-L2)	2	13	Total number of kicks in the endurance time test	Number of kicks (times)	a-tDCS improved the number of kicks in the endurance time test compared to sham
Yang et al. (2024)	Single-blind, RCT	24(6F,18M)	Professional swimmer	A—M1(C3,C4) C—M1(C3,C4) Halo Sport	2	20	400-m race TT	400 m (s)	a-tDCS improved Anaerobic endurance score compared to sham
Zhang et al. (2024)	Single-blind, crossover	29(9F,20M)	basketball player	A1— M1(C3,C4)A2—left M1(C3) C1—Bilateral shoulder C2—right M1(C4)	2	20	Shooting percentage test	Total number of field goals made (n)	a-tDCS improved the shooting percentage test compared to sham
Minghao and Zheng (2024)	Single-blind, RCT	28 (all M)	Volleyball player	A—M1(C3,) C—(FC3,CP3,C5,C1)	2	15	Serve speed test	Serve speed (m/h)	a-tDCS improved the serve speed test compared to sham
Jie et al. (2024)	Single-blind, RCT	30 (all M)	basketball player	A—left M1(C3) C—left superior orbit	2	20	The number of successful one-handed overhead shots from a fixed point	Number of Successful Shots (times)	a-tDCS improved the number of hits compared to sham
Yang (2024)	Single-blind, crossover	16 (all F)	Fencing athlete	A—M1(C3,) C—(FC1,FC5,CP5 、 CP1)	2	20	Stop Signal Task (SST)test	Reaction time (ms)	a-tDCS improved SST test compared to sham
Zhuangzhuang (2024)	Single-blind, RCT	24 (all M)	Sprinter	A—M1 C—Lateral shoulder	2	20	Standing Vertical Jump test	Vertical jump height (cm)	a-tDCS improved Standing Vertical Jump performance compared to sham
Rocha et al. (2024)	Triple-blind,crossover	18 (all M)	football player	A—M1(Cz) C—inion	2	15	CMJ	CMJ (cm)	No significant difference
Ramos et al. (2024)	Triple-blind,crossover	7 (all M)	rowing athlete	A—left TC C—Fp2	2	20	2 km rowing race time	2-km rowing time(s)	a-tDCS group achieved better 2 km rowing results than the sham stimulation group
Dai et al. (2024)	single-blind,crossover	16 (all F)	Fencing athlete	A—M1(C3,) C—(FC1,FC5,CP5,CP1)	2	20	RT	Reaction time (s)	No significant difference

Note: a-tDCS—Anodal Transcranial Direct Current Stimulation; BJT—Broad jump test; CB—Cerebellum; CMJ—Countermovement Jump; dlPFC—Dorsolateral Prefrontal Cortex; FSKT—Frequency Speed of Kicking Test; HD-tDCS—High-Definition Transcranial Direct Current Stimulation; M1—Primary Motor Cortex; PPO—Peak Power Output; PSTT—Progressive Taekwondo-Specific Test; TC—Temporal Cortex; TT—Time Trial; SEI—Short-Term Endurance Index; TTE—Time to Exhaustion; SST—Stop Signal Task Test.

## 3 Results

### 3.1 Study selection

**FIGURE 1:**
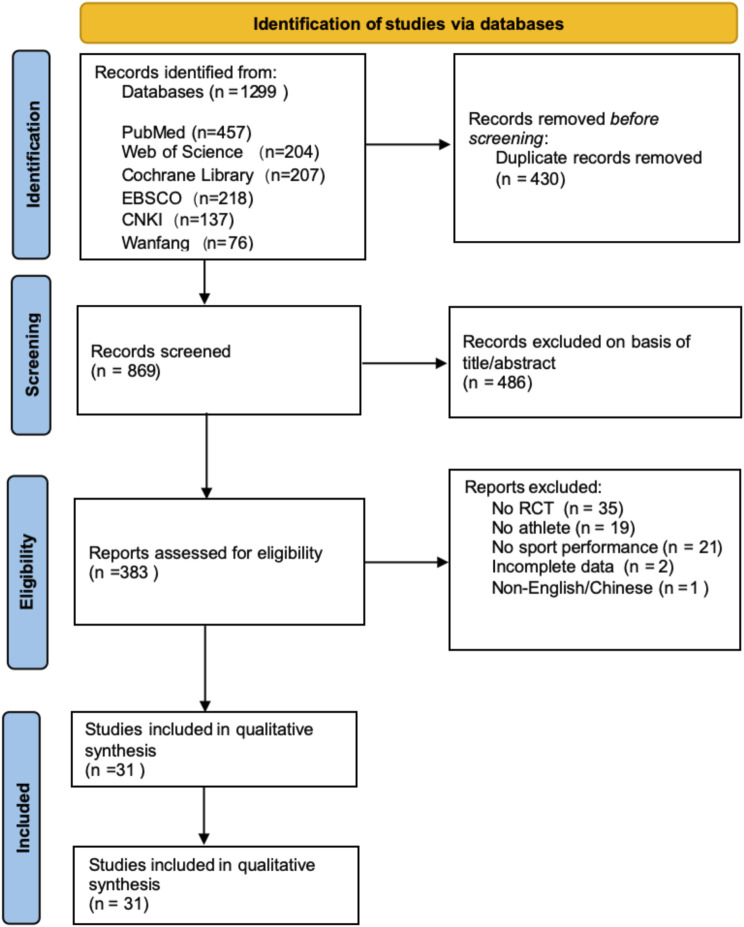
Literature Screening Flowchart (PRISMA declaration format).

Based on the search strategy, a total of 1,299 articles were retrieved. The retrieved literature was imported into Endnote 21 software, and 430 duplicate articles were removed. By reviewing abstracts and keywords, 486 articles irrelevant to the topic were excluded. Subsequently, 91 full-text articles were assessed for eligibility, and based on the PICOS criteria, 78 records were excluded for the following reasons: non-randomized controlled trials (n = 35), non-athlete participants (n = 19), no sport performance outcomes (n = 21), incomplete data (n = 2), and non-English/Chinese language (n = 1). Ultimately, 31 articles (25 in English, 6 in Chinese) meeting the inclusion criteria were included. The specific search process is illustrated in Figure 1.

### 3.2 Characteristics of included studies

Table 1 shows that all included studies were randomized controlled trials (RCTs) using sham stimulation as a control, published between 2015 and 2025, with the majority (65%) published in the last 3 years (2022–2024), highlighting recent interest in this research field. These studies collectively involved 473 athletes from various sports, including 331 males and 152 females. Sample sizes ranged from 8 to 30 participants. Athletes participated in diverse sports, including endurance sports (cycling, rowing, long-distance running), strength sports (bodybuilding, climbing, sprinting), precision skill-tasks (shooting, golf, gymnastics, fencing), and competitive and cooperative skill-based tasks (basketball, volleyball, taekwondo). Stimulation targeted primarily M1, PFC, TC, and CB, with all studies using tDCS prior to stimulation. Of these, 23 studies used conventional tDCS, 5 used Halo-Sport, and 3 used HD-tDCS, with current intensities ranging from 1 to 2.2 mA and stimulation durations from 10 to 30 min.

### 3.3 Quality assessment of included studies

**FIGURE 2:**
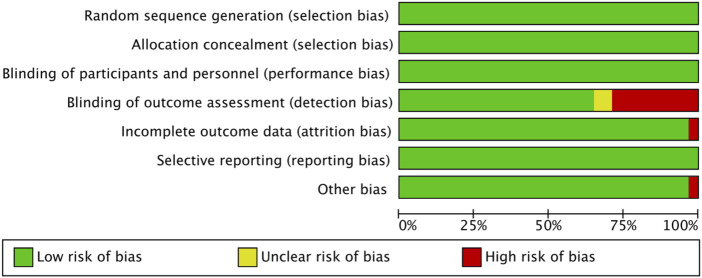
Risk of bias graph.

**FIGURE 3:**
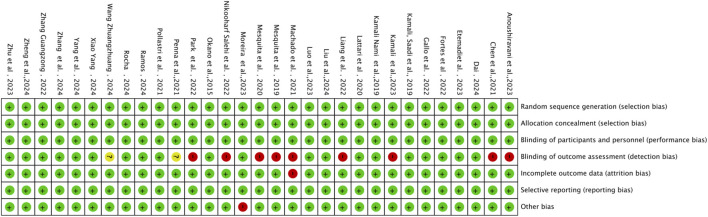
Risk of bias summary.

The methodological quality of the 31 included studies was generally high, with an average score of 6.56 points. Among them, 19 studies scored 7 points, 11 studies scored 6 points, and one study received a score of 5 (see Table 2). As illustrated in Figure 2, each item represents a type of potential bias, with risk levels denoted by color: green for low risk, yellow for unclear risk, and red for high risk. As Figure 2 indicates, most studies demonstrated low risk across most bias categories. However, some concerns remain, particularly regarding outcome assessor blinding, where a proportion of studies showed high or unclear risk. Figure 3 further details the distribution of risk assessments across each domain. Overall, the quality of the included literature can be considered relatively robust.

**TABLE 2:** Quality assessment table for included studies.

Study	Random sequence generation	Allocation concealment	Blinding of participants and personnel	Blinding of outcome assessment	Incomplete outcome data	Selective reporting	Other sources of bias	Score/ points
Okano et al. (2015)	+	+	+	+	+	+	+	7
Kamali et al. (2019b)	+	+	+	+	+	+	+	7
Kamali et al. (2019a)	+	+	+	+	+	+	+	7
Mesquita et al. (2019)	+	+	+	-	+	+	+	6
Lattari et al. (2020)	+	+	+	+	+	+	+	7
Mesquita et al. (2020)	+	+	+	-	+	+	+	6
Chen et al. (2021)	+	+	+	-	+	+	+	6
Pollastri et al. (2021)	+	+	+	+	+	+	+	7
Penna et al. (2021)	+	+	+	?	+	+	+	6
Machado et al. (2019b)	+	+	+	-	-	+	+	5
Nikooharf Salehi et al. (2022)	+	+	+	-	+	+	+	6
Liang et al. (2022)	+	+	+	-	+	+	+	6
Gallo et al. (2022)	+	+	+	+	+	+	+	7
Park et al. (2023)	+	+	+	-	+	+	+	6
Fortes et al. (2022)	+	+	+	+	+	+	+	7
Zhuangzhuang (2024)	+	+	+	+	+	+	+	7
Zhiqiang et al. (2023)	+	+	+	+	+	+	+	7
Moreira et al. (2023)	+	+	+	+	+	+	-	6
Luo et al. (2023)	+	+	+	+	+	+	+	7
Anoushiravani et al. (2023)	+	+	+	-	+	+	+	6
Etemadi et al. (2023)	+	+	+	+	+	+	+	7
Kamali et al. (2023)	+	+	+	-	+	+	+	6
Yang et al. (2024)	+	+	+	+	+	+	+	7
Zhang et al. (2024)	+	+	+	+	+	+	+	7
Minghao and Zheng (2024)	+	+	+	+	+	+	+	7
Jie et al. (2024)	+	+	+	+	+	+	+	7
Yang et al. (2024)	+	+	+	+	+	+	+	7
Zhuangzhuang et al. (2024)	+	+	+	?	+	+	+	6
Rocha et al. (2024)	+	+	+	+	+	+	+	7
Ramos et al. (2024)	+	+	+	+	+	+	+	7
Dai et al. (2024)	+	+	+	+	+	+	+	7

Note: Low risk of bias is indicated by “+”, high risk by “–”, and unclear risk by “?”. A score of 1 point is awarded for low risk of bias; no points are given for high or unclear risk.

### 3.4 Meta-analysis results

#### 3.4.1 Overall analysis

**FIGURE 4:**
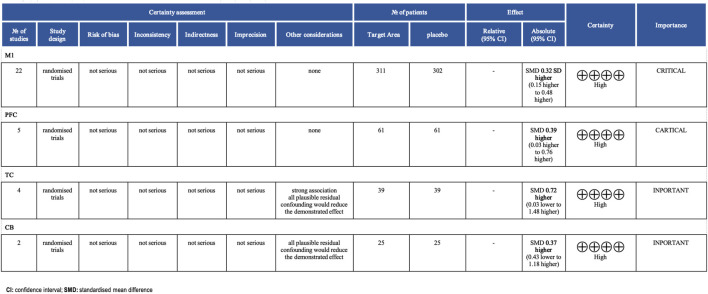
GRADE evidence profile.

**FIGURE 5:**
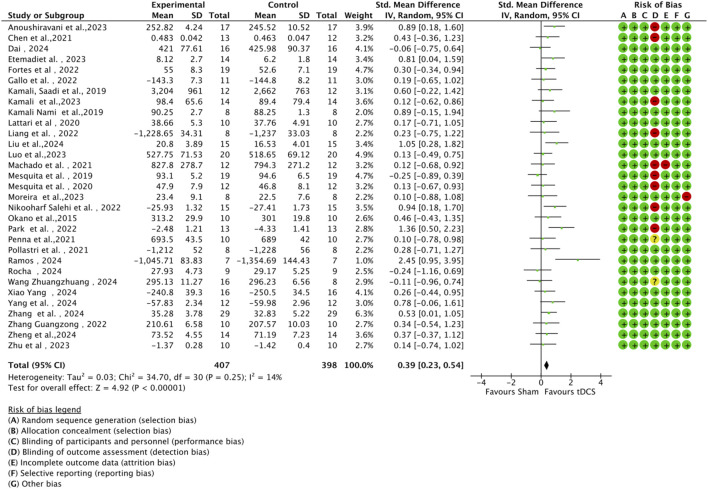
Forest plot of standardized mean differences (SMDs) for the overall effect of anodal tDCS on sport-specific motor performance relative to sham stimulation.

**FIGURE 6:**
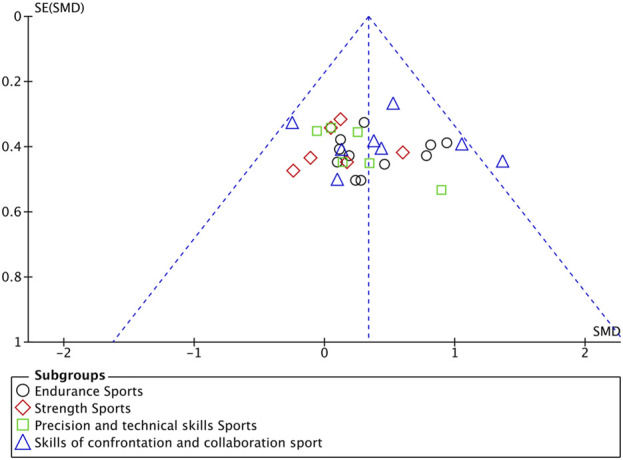
Funnel plot for publication bias.

In all included studies, a total of 407 participants in the experimental group and 398 participants in the control group were involved, evaluating the overall effect of tDCS on athletes’ sports performance. The GRADE summary table (Figure 4) includes data from 22 randomized trials for the M1 target area, 5 for the PFC target area, 4 for the TC target area, and 2 for the CB target area. There were no serious issues with risk of bias, inconsistency, indirectness, or imprecision, and the certainty assessment was rated as “high” for all. The forest plot (Figure 5) indicates a significant positive effect of a-tDCS on sports performance (n = 31, SMD = 0.39, 95% CI = 0.23 ∼ 0.54, p < 0.001), with low inter-study heterogeneity (I^2^ = 14%, χ^2^ = 34.70, p = 0.25). To verify the robustness of the results, a sensitivity analysis was conducted using a fixed-effect model. The funnel plot (Figure 6) shows the distribution of effect sizes and standard errors for each independent study; the graphical distribution reveals that most studies are clustered around the symmetry axis of the funnel plot, presenting a relatively balanced pattern with no obvious skewness, except for one effect size, with the remaining effect sizes falling within the funnel.

#### 3.4.2 Subgroup analysis by stimulation target area

**FIGURE 7:**
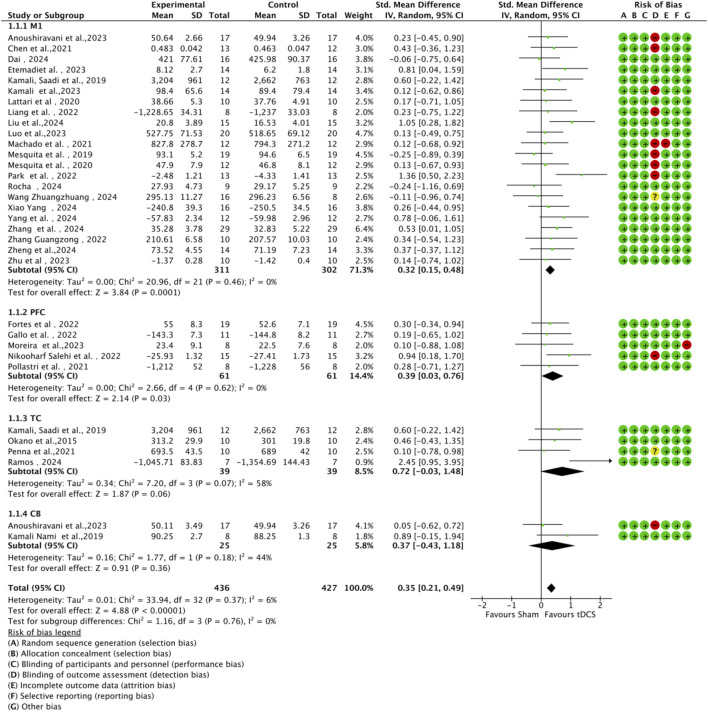
Funnel plot of included studies based on brain stimulation target area to assess publication bias.

A subgroup analysis was conducted based on different cortical stimulation targets (Figure 7). The results demonstrated that stimulation over the M1 and dorsolateral dlPFC yielded statistically significant effects. Specifically, stimulation of M1 (n = 22) produced a moderate and significant positive effect on athletic performance (SMD = 0.32, 95% CI = 0.15 ∼ 0.48, p < 0.001), highlighting its critical role in motor control. Similarly, stimulation of the PFC (n = 5) showed a moderate effect size (SMD = 0.39, 95% CI = 0.03 ∼ 0.76, p = 0.03), which may be attributed to its involvement in cognitive control and fatigue regulation. In contrast, the effects of stimulation over the TC (n = 4, SMD = 0.72, 95% CI = -0.03 ∼ 1.48, p = 0.06) and the CB (n = 2, SMD = 0.37, 95% CI = –0.43 ∼1.88, p = 0.36) did not reach statistical significance, likely owing to the limited number of included studies. Additionally, no significant differences were observed between subgroups (χ^2^ = 1.16, df = 3, p = 0.76), indicating comparable effect sizes across different target regions where significant results were obtained.

#### 3.4.3 Subgroup analysis by type of athletic performance

**FIGURE 8:**
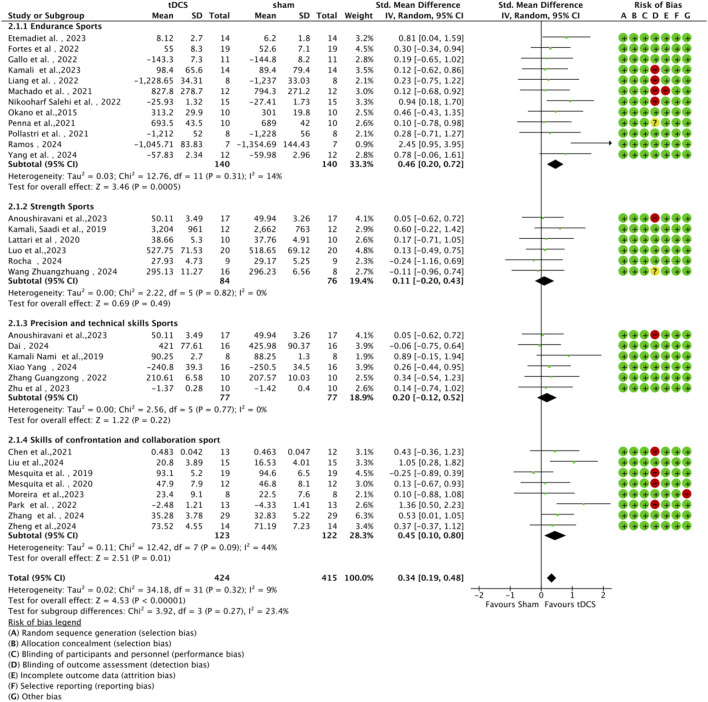
Forest plot showing subgroup analyses of the effects of anodal tDCS on different aspects of sport performance.

Studies were categorized into four subgroups based on the type of athletic performance: endurance, strength, precision skill tasks, and competitive-collaborative skill tasks (Figure 8). A statistically significant moderate effect (n = 12, SMD = 0.46, 95% CI = 0.20 ∼ 0.72, p < 0.001) was observed for endurance-related tasks, indicating that a-tDCS can effectively enhance sustained performance capacity. A significant positive effect was also demonstrated for interactive cooperative skill-based tasks (n = 8) such as basketball shooting and volleyball spiking, (n = 8, SMD = 0.45, 95% CI = 0.10 ∼ 0.80, p = 0.01). However, the effect sizes for strength tasks (n = 6, SMD = 0.11, 95% CI = -0.20 ∼ 0.43, p = 0.49) and precision skill tasks (n = 6, SMD = 0.20, 95% CI = -0.12 ∼ 0.52, p = 0.22) were not statistically significant. These non-significant findings may be attributed to potential ceiling effects in highly trained athletes or to the task-specific neural requirements that limit responsiveness to stimulation. Subgroup difference analysis did not reveal any statistically significant between-group differences (χ^2^ = 3.92, df = 3, p = 0.27), suggesting comparable effect magnitudes across performance types.

#### 3.4.4 Subgroup analysis by stimulation parameters

**FIGURE 9:**
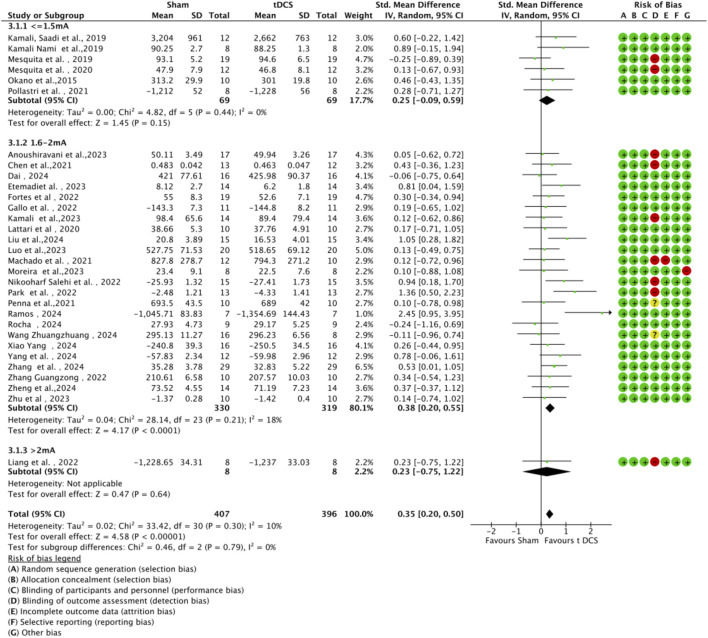
Forest plot of a-tDCS effects on motor performance by current intensity (low: ≤1.5 mA, medium: 1.6–2.0 mA, high: >2.0 mA).

**FIGURE 10:**
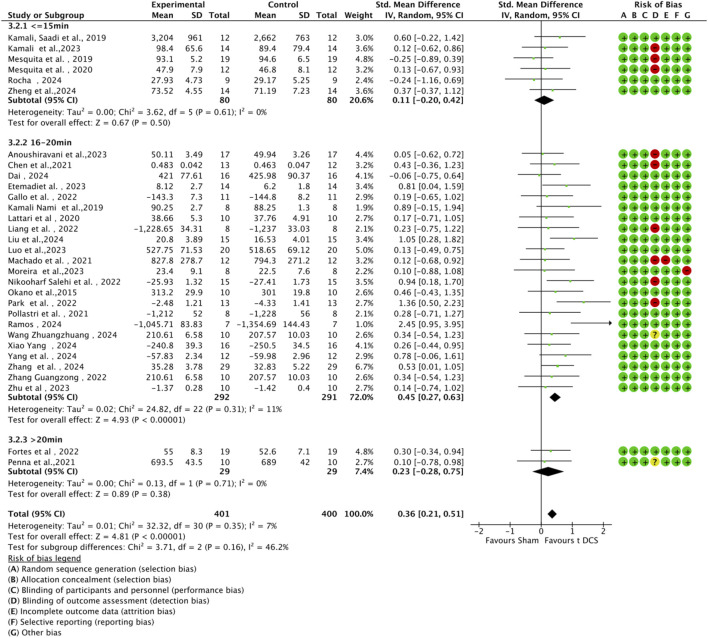
Forest plot of a-tDCS effects on motor performance by stimulation duration (short: ≤15 min, medium: 16–20 min, long: >20 min).

Subgroup analyses were conducted based on tDCS protocol parameters (current intensity and stimulation duration) to explore their impact on sports performance (Figures 9, 10). Regarding current intensity, studies were divided into three groups: low intensity (≤1.5 mA, n = 6), medium intensity (1.6–2.0 mA, n = 24), and high intensity (>2.0 mA, n = 1). The medium intensity group showed a significant effect (SMD = 0.38, 95% CI:0.42 ∼ 0.78, p < 0.001) compared to the high intensity group (SMD = 0.23, 95% CI: 0.75 ∼ 1.22, p < 0.64) and the low intensity group (SMD = 0.25, 95% CI: 0.09 ∼ 0.59, p = 0.15). For stimulation duration, studies were categorized into short duration (≤15 min, n = 6), medium duration (16–20 min, n = 23), and long duration (>20 min, n = 2). The medium duration group exhibited a significant effect (SMD = 0.45, 95% CI:0.27 ∼ 0.63, p < 0.001) compared to the short duration group (SMD = 0.11, 95% CI: 0.20 ∼ 0.42, p = 0.50) and the long duration group (SMD = 0.23, 95% CI: 0.28 ∼ 0.75, p = 0.38). Within each subgroup, heterogeneity was low, and there were no significant differences between subgroups (current intensity: χ^2^ = 0.46, df = 2, p = 0.79; duration: χ^2^ = 3.71, df = 2, p = 0.16).

## 4 Discussion

This meta-analysis aims to systematically evaluate the acute effects of a-tDCS on athletes’ motor performance. The results show that a-tDCS significantly improves motor performance, based on 31 randomized controlled trials, with particularly notable effects in endurance and competitive-collaborative skill tasks. Subgroup analysis confirms M1 as the most effective target area, followed by PFC, with a current intensity of 1.6–2.0 mA and a stimulation duration of 16–20 min as the optimal parameters. Next, we attempt to elucidate the underlying mechanisms of these effects.

The study found that a-tDCS significantly enhances performance in endurance and competitive-collaborative skill tasks, likely due to the synergistic effects of M1 and PFC stimulation. As a key hub for motor output, M1 optimizes motor unit recruitment by reducing action potential thresholds and enhancing corticospinal excitability, thereby extending time to exhaustion in endurance tasks (e.g., cycle ergometer tests) (Etemadi et al., 2023; Bhattacharjee et al., 2021). Additionally, M1 stimulation promotes motor memory formation through long-term potentiation (LTP)-like mechanisms, facilitating learning and retention in repetitive skill tasks (Nitsche and Paulus, 2000; Suppa et al., 2016). Research indicates that pain tolerance induced by exercise affects endurance performance (Astokorki and Mauger, 2017), and M1 stimulation may improve performance by alleviating exercise-induced pain (Gandevia, 2001; Taylor et al., 2016). Furthermore, PFC (particularly dlPFC) stimulation likely enhances cognitive control (Friedman and Robbins, 2022) and suppresses fatigue perception (Rupp and Perrey, 2008), improving athletes’ persistence under high-intensity or mental fatigue conditions (Angius et al., 2019). For competitive-collaborative skill tasks, a-tDCS may enhance neural plasticity in the M1-PFC network, improving perceptual-motor integration and movement execution accuracy. These mechanisms suggest that a-tDCS not only boosts physiological performance but also optimizes cognitive and psychological factors, particularly in competitive settings requiring sustained effort or complex coordination. The TC, a critical region for integrating auditory, language, and motor planning, plays a key role in advanced regulation of cardiac autonomic function (Dono et al., 2020; Reisert et al., 2021; Jackson et al., 2018). However, its deeper cortical location and the distance from scalp to cortex, coupled with tissue impedance, may limit tDCS current penetration, resulting in insufficient stimulation intensity to induce significant neural excitability changes. Similarly, the CB is central to coordination, motor prediction, sensory feedback integration, and balance maintenance, closely linked to various motor skills, postural control, and movement precision (Buckner, 2013; Sokolov et al., 2017). Notably, tDCS efficacy is constrained by the cerebellum’s anatomical structure and current conduction pathways, making it challenging for stimulation currents to penetrate the cerebellar cortex (Koziol et al., 2014; Verduzco-Flores and O'Reilly, 2015). Additionally, athletes’ baseline cortical excitability or training levels may influence CB responses to tDCS, potentially explaining the non-significant effects of TC and CB stimulation in subgroup analyses.

In terms of task types, the meta-analysis indicates that a-tDCS significantly enhances performance in endurance and competitive-collaborative skill tasks, while its effects on strength and precision skill tasks are not significant. The improvement in endurance tasks likely stems from M1 and PFC stimulation, which delays fatigue perception and enhances central drive (Machado et al., 2019b). These tasks involve prolonged low-intensity exercise, where neural factors such as pain tolerance and movement economy significantly influence performance. Competitive-collaborative skill tasks (e.g., basketball shooting, volleyball spiking) rely on fine motor control and perceptual-motor integration, with M1 stimulation significantly improving execution precision by enhancing neural plasticity and the efficiency of motor program retrieval. However, the effects of tDCS on athletic performance are not always positive. For instance, strength performance primarily depends on instantaneous muscle force generation, and while a-tDCS can enhance M1 cortical excitability, its direct impact on peripheral muscle fibers is limited, particularly in elite athletes where short-term stimulation struggles to overcome performance bottlenecks (da Silva Machado et al., 2021). The non-significant effect on precision skill tasks may be attributed to the complexity and diverse neural demands of these tasks. For example, tasks like shooting and golf require greater cognitive control and sensory integration than pure motor output, and single-target stimulation may be insufficient to comprehensively optimize performance.

The analysis of stimulation parameters indicates that a moderate current intensity of 1.6–2.0 mA and a moderate stimulation duration of 16–20 min represent the optimal protocol for enhancing motor performance. Low-intensity and short-duration stimulation yield weak effects, likely due to insufficient current to induce adequate cortical excitability or insufficient time to establish stable neural plasticity (Jamil et al., 2017). While high-intensity and long-duration stimulation perform better than low-intensity/short-duration protocols, they are less effective than moderate parameters, possibly due to excessive intensity causing cortical competition or prolonged stimulation leading to reduced neural adaptability. These findings suggest that moderate current intensity and duration effectively balance the induction of neural excitability and plasticity.

Compared with previous meta-analyses, this study establishes a more comprehensive evidence base by incorporating both Chinese and English literature (6 Chinese, 25 English studies), expanding to 31 studies (n = 473), and integrating the latest evidence up to March 2025. Angius Luca (Angius et al., 2018b) conducted a narrative review exploring the effects of tDCS on motor performance in healthy individuals but did not focus on athletes. Holgado (Holgado et al., 2019) analyzed the impact of tDCS on objective and subjective motor performance indicators but was limited by a smaller sample size and high heterogeneity. Holgado (Holgado et al., 2024) provided a broader umbrella review but lacked detailed subgroup analyses. (Maudrich et al., 2022) et al. emphasized the enhancement of sport-specific performance through single-session a-tDCS but did not include Chinese literature or thoroughly explore parameter optimization. The novelty of this study lies in: 1) being the first to integrate Chinese and English literature, reducing cultural and publication biases; 2) conducting refined subgroup analyses by brain region and dosage, confirming the optimal efficacy of M1 stimulation (1.6–2.0 mA, 16–20 min) for endurance and competitive-collaborative skill tasks; and 3) incorporating new studies to address gaps in prior research. Furthermore, the GRADE assessment (Figure 4) confirms high-certainty evidence for M1 and PFC stimulation, providing robust support for the practical application of tDCS.

Although this study provides high-certainty evidence for the impact of a-tDCS on athletic performance, several limitations remain. First, including only Chinese and English literature may overlook studies in other languages, limiting global representativeness. Second, small sample sizes in individual studies may reduce statistical power, particularly in TC and CB subgroup analyses, affecting result robustness. Third, heterogeneity in tDCS protocols (e.g., electrode placement, stimulation timing, athlete training levels) may influence effect consistency. Fourth, the focus on acute a-tDCS effects restricts inferences about long-term or repeated stimulation. Finally, athletes’ baseline cortical excitability or training levels (e.g., elite vs. amateur) may affect tDCS responsiveness, but data limitations prevented deeper exploration. These limitations suggest cautious interpretation of a-tDCS’s broad applicability. Future research should: 1) include multilingual literature to enhance global representativeness; 2) conduct large-scale, multi-center trials to improve statistical power; 3) standardize tDCS protocols (e.g., electrode placement, timing) to reduce variability; 4) explore cumulative effects of long-term tDCS; 5) develop personalized protocols to address individual athlete differences and validate a-tDCS’s practical value in real competition settings. These directions will further advance the optimized application of a-tDCS in athletic training.

Coaches may use a-tDCS (1.6–2.0 mA, 16–20 min, targeting M1 or dlPFC) as a pre-competition intervention to enhance endurance performance (e.g., prolonging time to exhaustion) or competitive-collaborative skills. However, the moderate certainty for strength and precision skill tasks suggests cautious application, recommending multi-target stimulation or higher-intensity protocols to further validate efficacy. The moderate effect size and acute nature indicate that coaches should temper expectations, integrating a-tDCS with conventional training to maximize benefits rather than relying solely on it.

## 5 Conclusion

The meta-analysis results show that the acute effects of a-tDCS significantly improve athletes’ endurance and competitive-collaborative skill performance by enhancing cortical excitability and neuroplasticity in M1 and PFC, with optimal stimulation parameters of 1.6–2.0 mA for 16–20 min. The effects on strength and precision skill tasks are limited. Future research should optimize stimulation strategies and incorporate neuroimaging techniques to enhance the application value of a-tDCS in competitive training.

## Data Availability

The raw data supporting the conclusions of this article will be made available by the authors, without undue reservation.
